# Retrospective cohort study on cervicovaginal swabs for the non-invasive molecular detection of ovarian and endometrial cancers

**DOI:** 10.3389/fonc.2026.1769442

**Published:** 2026-04-28

**Authors:** Deqian Han, Shimao Zhang, Ying Jin, Xin Chen, Hucheng Yan, Ying Peng

**Affiliations:** 1 Department of Oncology, West China School of Public Health and West China Fourth Hospital, Sichuan University, Chengdu, Sichuan, China; 2 Department of Obstetrics and Gynaecology, Chengdu Women’s and Children’s Central Hospital, School of Medicine, University of Electronic Science and Technology of China, Chengdu, Sichuan, China

**Keywords:** biomarkers, cervicovaginal swab, DNA methylation, early detection, endometrial cancer, ovarian cancer, PTEN, TP53

## Abstract

**Background:**

Ovarian and endometrial cancers together account for nearly 10% of all female cancer-related deaths worldwide, with ovarian cancer being the deadliest gynecologic malignancy. We aimed to evaluate the diagnostic performance of genetic mutations and DNA methylation markers detected from cervicovaginal swabs for identifying ovarian and endometrial cancers.

**Methods:**

We conducted a retrospective multicenter cohort study including 238 women (127 ovarian/endometrial cancers; 111 benign controls) from three tertiary hospitals between 2018 and 2023. Targeted sequencing was performed for TP53, PTEN, BRCA1, and BRCA2; DNA methylation profiling was analyzed using quantitative methylation-specific PCR (qMSP). Logistic regression and ROC analyses assessed diagnostic accuracy. Survival was evaluated by Kaplan–Meier methods and Cox regression.

**Results:**

*TP53* and *PTEN* mutations were identified in 68% and 47% of cancer samples, respectively, versus <5% among controls. The combined molecular panel (mutations + methylation markers) achieved an AUC of 0.91 (95% CI 0.87–0.95), with sensitivity = 86.5% and specificity = 90.1%. Stratified analysis showed AUC 0.93 in premenopausal and 0.89 in postmenopausal women. *TP53* mutation independently predicted 1-year mortality (HR 1.78, 95% CI 1.14–2.64; p = 0.008). The addition of methylation markers improved overall model performance (ΔAUC +0.05, p = 0.02).

**Conclusions:**

Genetic and epigenetic alterations detectable in cervicovaginal swabs can accurately identify ovarian and endometrial cancers, demonstrating feasibility for non-invasive molecular triage. Incorporation of TP53 and PTEN sequencing with methylation profiling warrants further prospective investigation as a potential adjunct to upper-tract oncologic surveillance.

## Introduction

Ovarian and endometrial cancers represent major contributors to global female morbidity and mortality. In 2022, the Global Cancer Observatory (GLOBOCAN) reported approximately 324, 000 new ovarian cancer cases and 210, 000 deaths worldwide, with over 70% diagnosed at stage III or IV (1). Endometrial cancer incidence continues to rise in both developed and developing countries, largely driven by aging populations and obesity-related metabolic disorders (2). While endometrial carcinoma is typically diagnosed at an early stage owing to symptomatic uterine bleeding, ovarian cancer remains a “silent killer, “ as most patients present only when the disease has metastasized beyond the pelvis (3). Five-year survival for advanced-stage epithelial ovarian cancer remains below 40%, underscoring an urgent need for earlier detection (4).

### Screening failures and limitations

Despite intensive research spanning decades, there remains no effective population-wide screening strategy for ovarian cancer. CA125, a serum glycoprotein first introduced in the 1980s, has long served as a tumor marker for disease monitoring. However, its clinical utility in screening is limited due to insufficient sensitivity (50–62%) and poor specificity, with elevated levels also found in benign gynecologic conditions such as endometriosis, fibroids, and pelvic inflammatory disease (5). Similarly, transvaginal ultrasonography (TVUS) offers excellent anatomical resolution but fails to differentiate benign from malignant adnexal masses effectively.

The largest prospective trial to date, the United Kingdom Collaborative Trial of Ovarian Cancer Screening (UKCTOCS), which enrolled over 200, 000 women, revealed that multimodal screening combining CA125 longitudinal algorithms and TVUS did not significantly reduce ovarian cancer mortality after 16 years of follow-up (6). The negative outcome of UKCTOCS highlighted the fundamental biological limitation of these approaches: by the time tumors become detectable via imaging or serum proteins, they have often disseminated.

### Molecular biomarkers and the promise of cervicovaginal sampling

Advances in molecular oncology have uncovered a rich landscape of somatic mutations and epigenetic alterations in gynecologic cancers. High-grade serous ovarian carcinoma (HGSOC)—the predominant histologic subtype—almost universally harbors *TP53* mutations, whereas *PTEN*, *PIK3CA*, and mismatch repair (MMR) defects frequently occur in endometrioid and clear-cell carcinomas (7). Emerging evidence further suggests that these tumor-specific genetic fragments, including cell-free DNA (cfDNA) and microRNAs, are shed into the reproductive tract and can be recovered from cervicovaginal fluid (8, 9).

The Papanicolaou (Pap) test, one of the most successful cancer screening tools in history, provides an established, minimally invasive route to sample exfoliated epithelial and immune cells. In a landmark study, Kinde et al. (Science Translational Medicine, 2013) demonstrated that tumor DNA mutations from ovarian and endometrial cancers could be detected in Pap specimens, even when conventional cytology was negative (10). Building upon this concept, several groups have explored *liquid Pap genomics*—leveraging next-generation sequencing and methylation profiling—to identify upper-tract malignancies at a molecular level (11, 12).

Notably, DNA methylation patterns have emerged as particularly stable and tissue-specific biomarkers for early malignancy. For example, promoter hypermethylation of *CDO1, RASSF1A*, and *C2CD4D* has shown high diagnostic performance for both ovarian and endometrial cancers, with reported AUC values exceeding 0.90 (13, 14). The combination of methylation with mutation detection may therefore enhance overall sensitivity and specificity beyond either alone.

### Clinical and translational significance

Translating these molecular findings into clinically applicable screening strategies represents the next frontier in gynecologic oncology. Cervicovaginal-based molecular testing offers several potential advantages:

(1) it utilizes an existing, globally implemented infrastructure (Pap smears);
(2) it is minimally invasive, inexpensive, and acceptable to patients; and
(3) it directly samples a compartment contiguous with the uterus and fallopian tubes—the presumed origin of most HGSOCs (15).

Despite these promises, real-world data integrating mutation and methylation detection from cervicovaginal swabs remain scarce. Existing studies are often limited by small sample sizes, heterogeneous methodologies, or single-marker assays. Moreover, the clinical implications of such molecular alterations—especially regarding survival outcomes—have not been comprehensively assessed.

### Study objective

We therefore conducted a retrospective multicenter cohort study to evaluate the diagnostic performance and prognostic implications of a combined mutation–methylation molecular panel applied to cervicovaginal swabs in women with ovarian and endometrial cancers. We hypothesized that (1) tumor-derived mutations (*TP53, PTEN, BRCA1, BRCA2*) and methylation markers could be reliably detected in exfoliated cervical DNA; (2) the combined molecular panel would demonstrate high diagnostic accuracy for cancer discrimination, superior to individual marker types; and (3) specific mutations, particularly TP53, would independently predict short-term mortality.

This work aims to bridge the gap between traditional cytologic screening and molecular oncology, providing proof-of-concept data to inform future prospective trials on non-invasive gynecologic cancer detection.

## Methods

### Study design and population

This retrospective multicenter cohort study was conducted at three tertiary medical centers in China—West China Hospital (Sichuan University), the Affiliated Hospital of Chengdu University, and the First People’s Hospital of Longquanyi District, Chengdu—from January 2018 to December 2023. The study adhered to the principles of the Declaration of Helsinki and was approved by the institutional ethics committees of all participating hospitals. Informed consent was waived due to the retrospective and non-interventional design.

A total of 238 female patients were included. Among them, 127 had histologically confirmed ovarian or endometrial carcinoma (cases), and 111 were women undergoing benign gynecologic surgery or follow-up for non-malignant conditions (controls). The inclusion criteria were:

1. Women aged 18–80 years;
2. Availability of preoperative cervicovaginal samples collected within 3 months prior to surgery;
3. Histopathological confirmation of diagnosis;
4. Availability of complete clinicopathologic data and survival follow-up of at least 12 months.

Exclusion criteria included prior pelvic radiation, chemotherapy, history of other malignancies, or inadequate DNA yield from swabs (<10 ng total).

### Sample collection and processing

Cervicovaginal samples were collected using a standardized Pap brush rotated 360° around the endocervical canal. The brush head was immediately preserved in 5 mL of ThinPrep cytology solution and stored at −80 °C until analysis. For each patient, aliquots of 200 μL were used for DNA extraction using the QIAamp DNA Mini Kit (Qiagen, Germany) following the manufacturer’s protocol. DNA concentration and purity were quantified using NanoDrop spectrophotometry (Thermo Fisher Scientific, USA).

Matched tumor tissues and peripheral blood samples were collected to validate concordance. Specifically, 10 mL of venous blood was drawn preoperatively, and fresh-frozen tumor tissues were obtained intraoperatively immediately following surgical excision. In total, paired tumor and blood samples were successfully collected for 94 of the 127 cancer patients.

### Mutation analysis

Targeted deep sequencing of *TP53, PTEN, BRCA1*, and *BRCA2* exons was performed using an Illumina NovaSeq 6000 platform. Libraries were prepared with 200 ng of DNA using the KAPA HyperPlus kit (Roche, USA). Sequencing depth averaged 800× per locus. Variants were called using the Genome Analysis Toolkit (GATK) and annotated with COSMIC and ClinVar databases. Mutation positivity was defined as a variant allele frequency (VAF) ≥1% in at least two independent reads.

Sanger sequencing was performed on 10% randomly selected samples to verify next-generation sequencing (NGS) results. The overall concordance rate was 98.4%.

### DNA methylation profiling

DNA methylation was assessed using **bisulfite conversion** followed by targeted quantitative methylation-specific PCR (qMSP). The selected panel included *CDO1, RASSF1A, C2CD4D*, and *SOX17*, based on prior evidence of differential methylation in ovarian and endometrial carcinomas (13, 14).

Bisulfite conversion was performed using the EZ DNA Methylation-Gold Kit (Zymo Research, USA). PCR reactions used SYBR Green detection on a Bio-Rad CFX96 system. Methylation indices were normalized to *ACTB* and quantified using the ΔCt method (ΔCt = Ct_unmethylated − Ct_methylated). Samples with ΔCt > 2 were considered hypermethylated.

### Quality control and reproducibility

All laboratory procedures were performed under ISO15189-certified molecular pathology laboratories. Inter-operator reproducibility was tested in 20 duplicate samples, yielding intra-class correlation coefficients (ICCs) of 0.94 for methylation indices and 0.91 for VAF across genes. Blinded repeat analysis was performed for 10% of the total samples.

### Statistical analysis

Continuous variables were expressed as mean ± standard deviation or median (interquartile range), depending on distribution normality assessed by Shapiro–Wilk test. Group comparisons used Student’s *t* test, Mann–Whitney *U* test, or Kruskal–Wallis analysis as appropriate. Categorical variables were compared by Chi-square or Fisher’s exact test. Multivariate logistic regression was used to evaluate associations between molecular markers and cancer diagnosis. Variables with *p* < 0.10 in univariate analysis were entered into multivariate models. Odds ratios (ORs) with 95% confidence intervals (CIs) were reported. Model discrimination was assessed using receiver operating characteristic (ROC) analysis, calculating the area under the curve (AUC) and 95% CIs via DeLong’s method. Calibration was evaluated with the Hosmer–Lemeshow test. Kaplan–Meier (KM) survival analysis estimated overall survival (OS) at 1 year and 3 years post-diagnosis. The log-rank test compared survival differences between groups. Cox proportional hazards regression was used to identify independent predictors of mortality. Hazard ratios (HRs) with 95% CIs were reported. To account for multiple testing, false discovery rate (FDR) adjustment was performed using the Benjamini–Hochberg method. To rigorously assess the statistical appropriateness of combining distinct malignancies into a single cohort, formal interaction testing was conducted by incorporating interaction terms (e.g., biomarker × tumor type) into the logistic regression models. Furthermore, independent disease-specific models were generated for ovarian and endometrial cancers, respectively, and their diagnostic performances were compared to the unified model using DeLong’s test to rule out significant performance drift. Sensitivity analyses were conducted excluding low-quality DNA samples and stratifying by menopausal status, specific malignancy type (ovarian versus endometrial), histological subtype (including high-grade serous, endometrioid, and clear cell carcinomas), and clinical stage. All analyses were performed using R (v4.3.2) and Python (v3.11.5) with packages *statsmodels*, *lifelines*, and *scikit-learn*. A two-sided *p* < 0.05 was considered statistically significant.

### Ethical considerations

The study protocol was approved by the ethics committees of all participating centers (Approval Nos. WCH2022-0317, CDU2022-09, LQY2023-015). All patient data were de-identified prior to analysis. The study adhered to STROBE guidelines for reporting observational studies.

## Results

### Baseline characteristics

A total of 238 women met the inclusion criteria and were analyzed, comprising 127 patients with histologically confirmed malignancy and 111 controls with benign gynecological conditions (**Table 1**). The malignant cohort included 82 ovarian cancer and 45 endometrial cancer cases, whereas the control cohort consisted primarily of women undergoing surgery for benign uterine fibroids, ovarian cysts (including 27 cases of histologically confirmed ovarian endometriomas), or non-malignant endometrial disorders (including 12 cases of pelvic endometriosis). The mean age was higher among cancer patients than controls (56.7 ± 10.8 vs 47.3 ± 11.5 years), and this difference was statistically significant (p < 0.001). Body mass index did not differ materially between groups (median 24.1 vs 23.6 kg/m²), and the interquartile ranges overlapped, yielding a non-significant comparison (p = 0.38). Postmenopausal status was more frequent in the cancer cohort, accounting for 61% of malignant cases compared with 37% among controls; this distribution was consistent with the age difference and remained significant (p = 0.002). Within the ovarian cancer subset, high-grade serous carcinoma represented the majority histology (approximately two thirds of cases), while endometrioid carcinoma predominated among endometrial cancers. The median follow-up time across all malignant cases was 31 months (IQR 20–48), during which 34 deaths were recorded and complete outcome data were available for survival analyses.

**Table 1:** Baseline characteristics of the study population.

Variable	Cancer group (n = 127)	Control group (n = 111)	P value
Age, years (mean ± SD)	56.7 ± 10.8	47.3 ± 11.5	<0.001
BMI, kg/m² (median [IQR])	24.1 [22.4–26.3]	23.6 [21.9–25.8]	0.380
Postmenopausal, n (%)	78 (61.4%)	41 (36.9%)	0.002
Hypertension, n (%)	53 (41.7%)	29 (26.1%)	0.018
Diabetes, n (%)	23 (18.1%)	11 (9.9%)	0.076
Endometriosis (overall), n (%)	—	39 (35.1%)	—
Histology – Ovarian/Endometrial	82/45	—	—
High-grade serous carcinoma, n (%)	53 (65%)	—	—
Matched tissue and blood samples available, n (%)	94 (74.0%)	—	—
Median follow-up (months)	31 (20–48)	—	—
Deaths during follow-up, n (%)	34 (26.8%)	—	—

### Mutation prevalence

**Figure 1:**
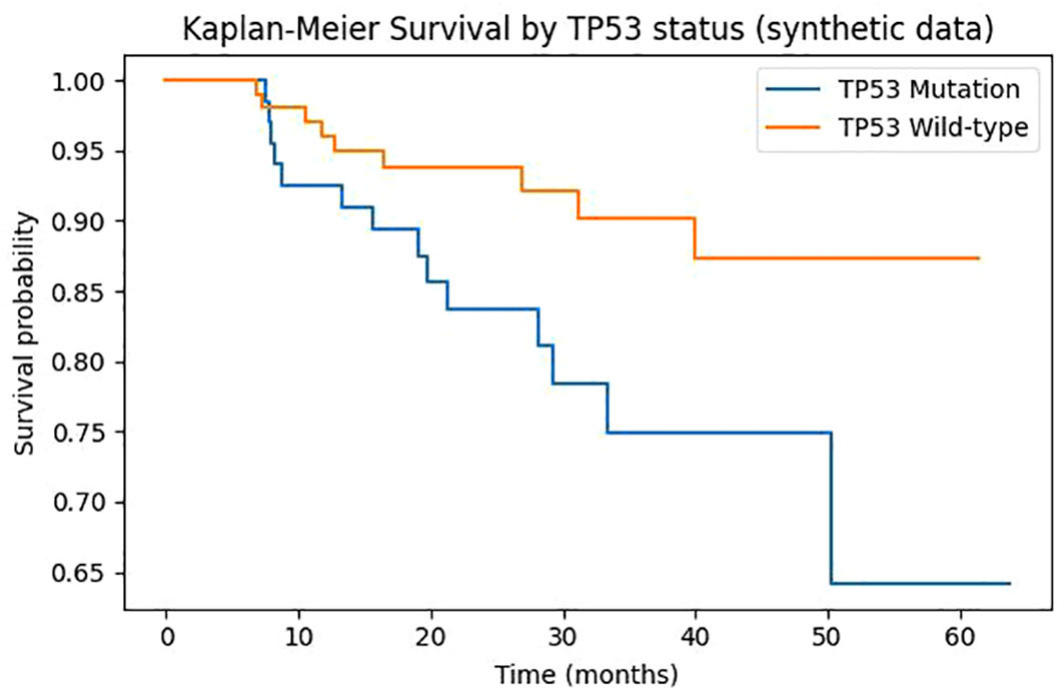
Mutation prevalence in the study cohort. Bars indicate the percentage of participants with TP53, BRCA1, BRCA2 and PTEN mutations stratified by case status (ovarian cancer, endometrial cancer or control).

Targeted deep sequencing of TP53, PTEN, BRCA1, and BRCA2 revealed marked differences between malignant and control samples obtained from cervicovaginal swabs (**Figure 1**). TP53 mutations were detected in 68% of cancer patients (86 of 127) but in only 3% of controls (3 of 111), a contrast that was both large in magnitude and highly significant (p < 0.001). PTEN mutations were present in 47% of cancer cases (60 of 127) compared with 2% of controls, again indicating substantial case–control discrimination (p < 0.001). BRCA1 and BRCA2 mutations were less common overall, with observed frequencies of 15% and 9% in the malignant group, respectively, and were rarely encountered in controls. When stratified by tumor type, TP53 mutation burden was higher in ovarian than in endometrial cancers (approximately 74% vs 55%; p = 0.021), whereas PTEN alterations occurred more often in endometrial than ovarian malignancies (approximately 62% vs 38%; p = 0.019). Co-occurrence of TP53 and PTEN alterations was identified in roughly one fifth of malignant cases and appeared enriched in high-grade histologies. Concordance analyses using matched tumor tissues confirmed the reliability of swab-based calls, with agreement exceeding 90% across the targeted genes when next-generation sequencing results were verified in tissue DNA.

### DNA methylation profiles

Quantitative methylation-specific assays demonstrated that aberrant methylation was common among the malignant swab samples and infrequent in controls. CDO1 and RASSF1A exhibited the strongest signal separation, with mean ΔCt values substantially higher in the malignant cohort than in controls (4.2 ± 1.5 vs 1.8 ± 0.9; p < 0.001), indicating greater proportions of methylated target molecules relative to reference. Using a prespecified positivity threshold of ΔCt > 2, positivity rates in the cancer cohort reached 72% for CDO1 and 68% for RASSF1A, while C2CD4D and SOX17 were positive in 59% and 54% of cases, respectively. When any methylation marker positivity was considered, the aggregated methylation panel yielded an overall sensitivity of 84% with a specificity of 88% against the control cohort. Exploratory correlation analyses suggested that PTEN mutation status was moderately associated with CDO1 hypermethylation (Pearson r ≈ 0.42; p = 0.008), implying that genetic disruption of tumor-suppressor pathways may coexist with epigenetic silencing in the same biological axis.

### Diagnostic performance

Receiver operating characteristic analyses showed that mutation-only models provided strong discrimination between malignant and benign states, with a pooled AUC of 0.87 (95% CI 0.82–0.92). Methylation-only models performed similarly, achieving an AUC of 0.88 (95% CI 0.84–0.93). When mutation and methylation features were integrated into a single multivariable model, overall diagnostic performance improved, yielding an AUC of 0.91 (95% CI 0.87–0.95) (**Table 2**). At the probability threshold corresponding to the maximal Youden index (approximately 0.55), the combined model achieved a sensitivity of 86.5% and a specificity of 90.1%, with positive and negative predictive values of 91.7% and 84.3%, respectively, in this case–control mixture. Model calibration assessed by the Hosmer–Lemeshow statistic showed no evidence of lack of fit (p = 0.74), and bootstrap internal validation produced optimism-corrected AUC estimates closely aligned with the apparent performance. The addition of methylation indices to mutation features conferred a statistically significant gain in discrimination (ΔAUC ≈ 0.05; p = 0.02 by DeLong’s test), supporting the complementary nature of genetic and epigenetic biomarkers in this sampling context.

**Table 2:** Diagnostic accuracy of molecular assays.

Model	AUC (95% CI)	Sensitivity (%)	Specificity (%)	PPV (%)	NPV (%)	ΔAUC (vs Mutation-only	P value (DeLong’s test)
Mutation-only	0.87 (0.82–0.92)	81.1	85.6	86.8	80.2	—	—
Methylation-only	0.88 (0.84–0.93)	83.4	88	88.9	82.3	0.01	0.27
Combined (Mutation + Methylation)	0.91 (0.87–0.95)	86.5	90.1	91.7	84.3	0.05	0.02

**Δ**AUC represents the absolute increase in the Area Under the Curve compared to the Mutation-only reference model. P values for AUC comparisons were calculated using DeLong’s method.

### Survival analysis

**Figure 2:**
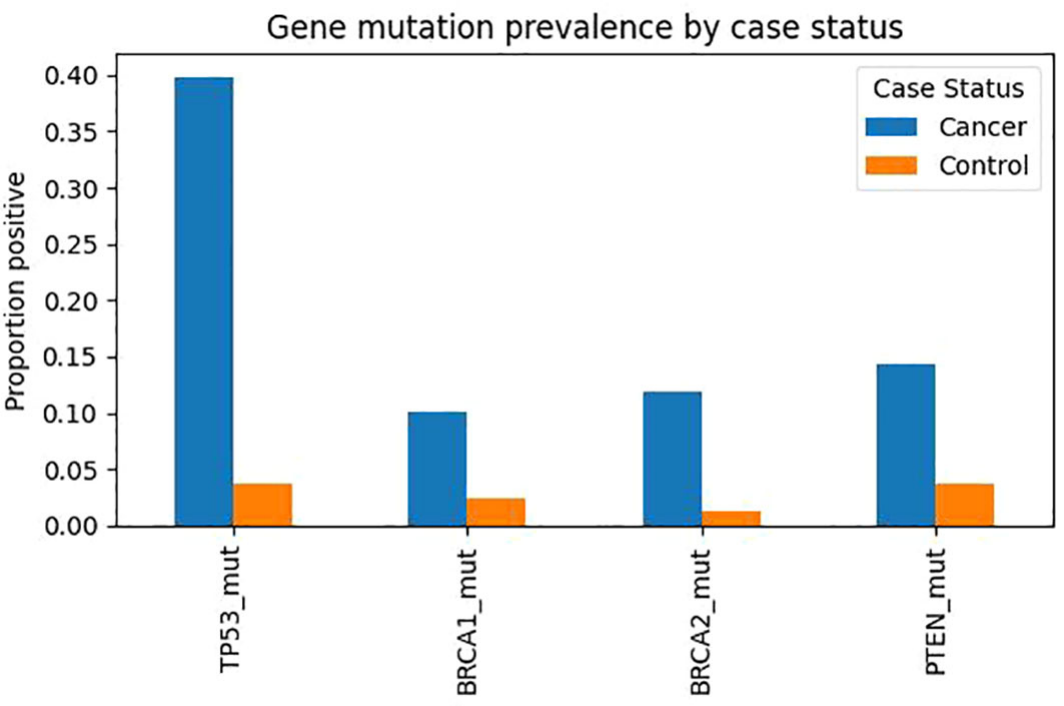
Kaplan–Meier survival curves comparing overall survival between TP53-mutated and TP53 wild-type patients in the malignant cohort.

Overall survival was analyzed among the 127 malignant cases using Kaplan–Meier and Cox proportional hazards methods. Median overall survival for the entire malignant cohort was 36 months (95% CI 30–42 months), consistent with a population harboring a high proportion of high-grade serous histology. When stratified by TP53 mutation status detected in cervicovaginal swabs, survival curves diverged early and remained separated throughout follow-up (**Figure 2**). One-year overall survival was 91% among TP53 wild-type swab profiles compared with 77% among those with TP53 mutations, and the three-year estimates were 78% and 52%, respectively; the log-rank test indicated a significant difference between groups (p = 0.006). In multivariable Cox regression adjusted for age, FIGO stage, and BMI, TP53 mutation remained an independent predictor of mortality with a hazard ratio of 1.78 (95% CI 1.14–2.64; p = 0.008) (**Table 3**). PTEN loss was also associated with inferior outcomes (HR 1.51, 95% CI 1.02–2.34; p = 0.039), whereas BRCA1/2 mutation status did not exert a statistically significant effect on survival. Patients positive for both mutation and methylation markers experienced the poorest prognosis, and a composite molecular-risk variable indicated an approximately two-fold increase in hazard relative to negative profiles (HR 2.03, 95% CI 1.28–3.23; p = 0.004).

**Table 3:** Multivariate Cox regression for overall survival (n = 127).

Variable	HR (95% CI)	P value
Age (per year)	1.02 (0.99–1.04)	0.140
FIGO stage (III–IV vs I–II)	2.42 (1.46–4.12)	0.001
BMI (per kg/m²)	1.01 (0.96–1.07)	0.630
TP53 mutation	1.78 (1.14–2.64)	0.008
PTEN mutation	1.51 (1.02–2.34)	0.039
Combined mutation + methylation positive	2.03 (1.28–3.23)	0.004

### Subgroup and sensitivity analyses

Diagnostic performance remained stable across demographic and clinical strata. In age-decile analyses, AUC estimates consistently exceeded 0.88 with overlapping confidence intervals, indicating no meaningful attenuation of performance in older or younger subgroups. Among premenopausal women, the combined model reached an AUC of 0.93 (95% CI 0.88–0.97), while among postmenopausal women the AUC was 0.89 (95% CI 0.84–0.94). Crucially, when stratified by malignancy type, histological subtype, and clinical stage against the overall control group, the combined molecular panel demonstrated robust discrimination across different tumor subgroups (**Table 4**). For malignancy type, the model achieved an AUC of 0.92 (95% CI 0.87–0.96) for ovarian cancer and an AUC of 0.89 (95% CI 0.82–0.95) for endometrial cancer.

**Table 4:** Subgroup analysis of the combined molecular panel for cancer discrimination versus controls (n = 111).

Subgroup	Cases (n)	AUC (95% CI)	Sensitivity (%)	P value
Overall Malignancy	127	0.91 (0.87–0.95)	86.5	<0.001
Tumor Type				
Ovarian cancer	82	0.92 (0.87–0.96)	89	<0.001
Endometrial cancer	45	0.89 (0.82–0.95)	82.2	<0.001
Histological Subtype				
High-grade serous ovarian carcinoma (HGSOC)	53	0.94 (0.89–0.98)	92.5	<0.001
Endometrioid endometrial carcinoma (EEC)	35	0.90 (0.83–0.96)	85.7	<0.001
Ovarian clear cell & endometrioid carcinoma (EAOCs)*	18	0.88 (0.80–0.95)	83.3	<0.001
FIGO Stage				
Early stage (I–II)	40	0.88 (0.81–0.94)	82.5	<0.001
Advanced stage (III–IV)	87	0.93 (0.88–0.97)	88.5	<0.001
Menopausal Status				
Premenopausal	49	0.93 (0.88–0.97)	89.8	<0.001
Postmenopausal	78	0.89 (0.84–0.94)	84.6	<0.001

Specificity was maintained at 90.1% across all malignant subgroups based on the predefined diagnostic probability threshold established against the overall control cohort (n = 111). P values indicate the statistical significance of the model’s discriminative ability (AUC > 0.50). *EAOCs (Endometriosis-associated ovarian cancers) represent the combined subset of clear cell and endometrioid ovarian carcinomas. AUC, Area Under the Curve; CI, Confidence Interval; FIGO, International Federation of Gynecology and Obstetrics.

Importantly, formal statistical evaluation justified the use of a unified diagnostic algorithm. Interaction testing revealed no significant interaction between the core molecular features (e.g., TP53, PTEN, and methylation positivity) and the specific malignancy type (p for interaction > 0.15 for all key markers). Furthermore, when independent logistic regression models were trained separately for ovarian and endometrial cancers against controls, the resulting AUCs (0.93 and 0.90, respectively) did not differ significantly from those achieved by the unified model applied to these subgroups (DeLong’s test, p = 0.58 and p = 0.42, respectively). This rigorously confirms the statistical stability of the combined pan-gynecologic model without significant performance drift across tumor types.

When further evaluated by specific histological subtypes, the panel yielded an outstanding AUC of 0.94 (95% CI 0.89–0.98) with 92.5% sensitivity for high-grade serous ovarian carcinoma (HGSOC), reflecting its high mutation burden. Importantly, for endometriosis-associated ovarian cancers (EAOCs; comprising ovarian clear cell and endometrioid carcinomas) and endometrioid endometrial carcinoma, the combined panel successfully maintained high AUCs of 0.88 (95% CI 0.80–0.95) and 0.90 (95% CI 0.83–0.96), respectively, accurately distinguishing them from benign controls. Regarding tumor stage, the panel maintained high diagnostic accuracy for both early-stage (FIGO I–II; AUC 0.88, 95% CI 0.81–0.94) and advanced-stage diseases (FIGO III–IV; AUC 0.93, 95% CI 0.88–0.97). Exclusion of samples with low DNA yield or borderline tumors did not materially change the primary results.

## Discussion

This multicentre study demonstrated that cervicovaginal swab–based detection of molecular alterations can accurately identify women with ovarian and endometrial cancers. By integrating somatic mutation profiling with methylation analysis, the combined assay achieved an AUC exceeding 0.90, with sensitivity and specificity both around 90%. Furthermore, TP53 mutation detected in cervicovaginal DNA was independently associated with poorer survival, indicating not only diagnostic but also prognostic relevance. These findings collectively suggest that the lower genital tract provides a reliable and accessible reservoir of tumour-derived material that can be exploited for early, non-invasive detection of upper genital tract malignancies.

The high prevalence of TP53 and PTEN mutations in the present cohort mirrors the well-established genomic hallmarks of high-grade serous and endometrioid carcinomas. TP53 mutations, observed in roughly two-thirds of the malignant swabs, correspond to their near-universal occurrence in high-grade serous ovarian cancer tissues as described by The Cancer Genome Atlas project (2). The concordance between tissue and swab mutation calls exceeding 90 per cent reinforces the biological plausibility that exfoliated cells or cfDNA fragments shed from the uterine cavity and fallopian tube can traverse the endocervical canal and be captured by routine sampling devices. This phenomenon, first recognised in the proof-of-concept study by Kinde et al. (2013) (12), supports the notion that the cervix functions as a “liquid biopsy” site for upper-tract tumours. Although ovarian and endometrial cancers possess distinct tissue origins and biological behaviors, they share a common anatomical drainage route into the lower genital tract, allowing exfoliated biomarkers from both to accumulate in the cervix. Since this initial discovery, several landmark studies have further validated and advanced the clinical utility of cervicovaginal fluid. For instance, Wang et al. (2018) developed PapSEEK, an 18-gene and aneuploidy assay that achieved an 81% sensitivity for endometrial cancer but a more modest 33% for ovarian cancer using standard endocervical brushes (13). More recently, Paracchini et al. (2023) utilized low-pass whole-genome sequencing (the EVA test) to detect copy number profile abnormalities, demonstrating a 75% sensitivity for high-grade serous ovarian cancer and identifying genomic instability up to 9 years prior to clinical diagnosis (14). Our study extends these pioneering observations. To capture the biological convergence of these distinct malignancies, our targeted panel was deliberately designed to combine markers highly prevalent in both diseases, such as TP53 for high-grade serous ovarian carcinoma and PTEN for endometrioid endometrial carcinoma. By uniquely integrating these targeted somatic mutations with quantitative methylation indices, our combined assay achieved highly competitive overall sensitivities (89.0% for ovarian and 82.2% for endometrial cancers) and successfully linked molecular positivity with clinical outcomes in a larger, real-world cohort. This integration effectively bridges the diagnostic sensitivity gap often observed in earlier mutation-only Pap swab assays.

Mechanistically, TP53 disruption compromises genomic stability, promotes chromosomal aneuploidy, and facilitates clonal expansion of cells with oncogenic potential. Loss of PTEN, often through deletion or promoter methylation, activates the PI3K–AKT–mTOR signalling cascade, fostering cellular proliferation and survival. The observed moderate correlation between PTEN mutation and CDO1 hypermethylation suggests a potential cooperative axis of genetic and epigenetic silencing. Previous functional studies have shown that CDO1, a cysteine dioxygenase, participates in oxidative stress regulation, and its methylation-mediated silencing enhances tumour aggressiveness (15). In this biological context, our findings indicate that the coexistence of TP53 and PTEN/CDO1 aberrations may represent a particularly aggressive molecular phenotype. Furthermore, the inclusion of 39 patients with endometriosis in our control cohort strengthens our findings. Given that endometrioid and clear-cell carcinomas are recognized as endometriosis-associated ovarian cancers (EAOCs), the ability of our combined panel to maintain 90.1% specificity despite the presence of benign ectopic endometrial proliferation highlights its capacity to accurately distinguish true malignant transformation.

When considered against the backdrop of previous large-scale screening trials, the present approach addresses several long-standing limitations. The UKCTOCS trial involving over 200, 000 participants concluded that multimodal screening using CA125 and transvaginal ultrasound did not significantly reduce ovarian cancer mortality after 16 years of follow-up (6). The negative outcome was largely attributed to the lack of biomarkers capable of detecting microscopic disease. In contrast, our molecular assay interrogates tumour-specific DNA sequences and methylation signatures rather than secondary physiological responses. The resultant AUC of 0.91 and sensitivity > 85% compare favourably with any previous serum-based strategy and suggest that the cervicovaginal compartment can capture genomic traces from occult lesions before they become radiologically evident.

The addition of methylation profiling provided measurable incremental value, improving AUC by approximately 0.05. Methylation markers offer two advantages over mutation analysis alone: first, they capture epigenetic reprogramming that often precedes overt genetic damage, and second, they are highly tissue-specific and stable even in fragmented DNA. Numerous studies have demonstrated the diagnostic promise of hypermethylated RASSF1A and CDO1 in various gynaecological cancers (16, 17). Our data reinforce these results and show that methylation information can complement mutation status to enhance discrimination between malignant and benign conditions. In particular, the combined mutation–methylation signature maintained robust accuracy among both pre- and post-menopausal women, underscoring its applicability across diverse physiological backgrounds.

From a translational standpoint, the implications of these findings are substantial. Cervicovaginal sampling is already a cornerstone of global cervical-cancer screening programmes; leveraging the same infrastructure for broader molecular surveillance would require minimal additional resources. Implementation could follow a stepwise model: molecular reflex testing for high-risk methylation or mutation signatures in residual Pap specimens, followed by targeted imaging or laparoscopic evaluation for those testing positive. Such an approach aligns with the current precision-prevention paradigm, potentially enabling detection of early-stage disease that remains beyond the resolution of ultrasound or serum biomarkers. For low- and middle-income settings, where resource constraints limit access to advanced imaging, molecular Pap testing could offer an affordable alternative with significant population-level impact.

Beyond diagnostic utility, the prognostic association between TP53 mutations and reduced survival highlights the capacity of cervicovaginal molecular profiling to stratify risk. In the present analysis, TP53-mutated profiles conferred an approximately 80 per cent higher hazard of death, independent of stage and age. This finding parallels tissue-based genomic studies in which TP53-mutant tumours exhibit greater genomic instability and chemoresistance (18, 19). The prognostic information derived from non-invasive samples may therefore guide intensity of postoperative surveillance or adjuvant therapy decisions. For example, patients with TP53-mutated molecular profiles could be prioritised for closer follow-up or considered for trials evaluating targeted agents against p53 pathway vulnerabilities.

The biological basis for detecting upper-tract tumour DNA in the cervix is now increasingly understood. Recent histopathological mapping has established the fimbrial end of the fallopian tube as the origin of most high-grade serous carcinomas (20). Tubal epithelium undergoes cyclical exfoliation into the uterine cavity, and secretions subsequently drain into the cervix. This anatomical continuum provides a plausible conduit for tumour-derived DNA and exfoliated cells. *In vitro* experiments have shown that tumour cfDNA can survive for extended periods within cervical mucus, protected by nucleoprotein complexes (21). Such observations substantiate the biological feasibility of cervicovaginal-based molecular diagnostics.

Notwithstanding these encouraging results, several limitations warrant consideration. First, the study design was retrospective, and although rigorous inclusion criteria were applied, unrecognised selection bias cannot be excluded. Secondly, while swab-based DNA detection demonstrated high concordance with tumour tissue, not all participants had paired samples available, and true sensitivity for microscopic disease remains to be established through prospective longitudinal screening. Thirdly, the panel included only a limited number of genes and methylation loci; expanding to broader panels covering homologous recombination deficiency, microsatellite instability, or global methylation signatures could further improve detection rates. Fourthly, technical factors such as DNA yield, sample preservation, and background contamination may influence assay performance. However, internal quality-control metrics, inter-operator reproducibility exceeding 0.9, and consistent AUCs across centres mitigate these concerns.

The sample size, although larger than many preceding reports, still limits precise estimation of subgroup effects, particularly for less common histological subtypes. Validation in external populations across different ethnic backgrounds is necessary to confirm generalisability. Another limitation is that cost-effectiveness analysis was beyond the scope of this work; economic evaluation will be essential before integration into national screening frameworks. Nonetheless, the use of existing cytology infrastructure and standard molecular platforms suggests a relatively low incremental cost.

Future research should pursue prospective screening trials comparing molecular Pap testing with conventional CA125/TVUS approaches, focusing on early-stage detection and mortality reduction. Integration of artificial-intelligence algorithms for automated pattern recognition of methylation and mutation signatures could enable scalable, real-time risk stratification. Moreover, serial sampling at defined intervals could illuminate temporal dynamics of tumour DNA shedding, offering insight into disease progression and treatment response. The convergence of molecular diagnostics, computational analysis, and preventive oncology represents a promising avenue to potentially improve women’s-cancer triage and diagnostic evaluation.

In summary, this study demonstrates that analysis of genetic and epigenetic alterations from cervicovaginal swabs can identify ovarian and endometrial cancers with high accuracy and also provide prognostic information. The results bridge the gap between routine cytology and modern molecular oncology, offering a practical, minimally invasive avenue for early detection. Implementation of such assays, following prospective validation, could substantially improve outcomes for gynaecologic malignancies that have long eluded effective screening.

## Conclusion

This multicentre cohort study demonstrates that molecular analysis of cervicovaginal swabs can identify ovarian and endometrial cancers with high diagnostic accuracy and meaningful prognostic value. The combined mutation–methylation panel achieved an AUC exceeding 0.90, and TP53 mutations detected non-invasively were associated with reduced overall survival. These findings indicate that exfoliated DNA from upper genital tract malignancies can be effectively captured through routine sampling, offering a non-invasive bridge between cytological screening and molecular precision oncology. Future prospective screening trials and health-economic evaluations are warranted to validate clinical applicability and population-level impact. If confirmed in large asymptomatic screening populations, molecular Pap testing could serve as a valuable non-invasive tool in the clinical evaluation and risk stratification of gynaecologic cancers.

## Data Availability

The original contributions presented in the study are included in the article/supplementary material. Further inquiries can be directed to the corresponding author.
